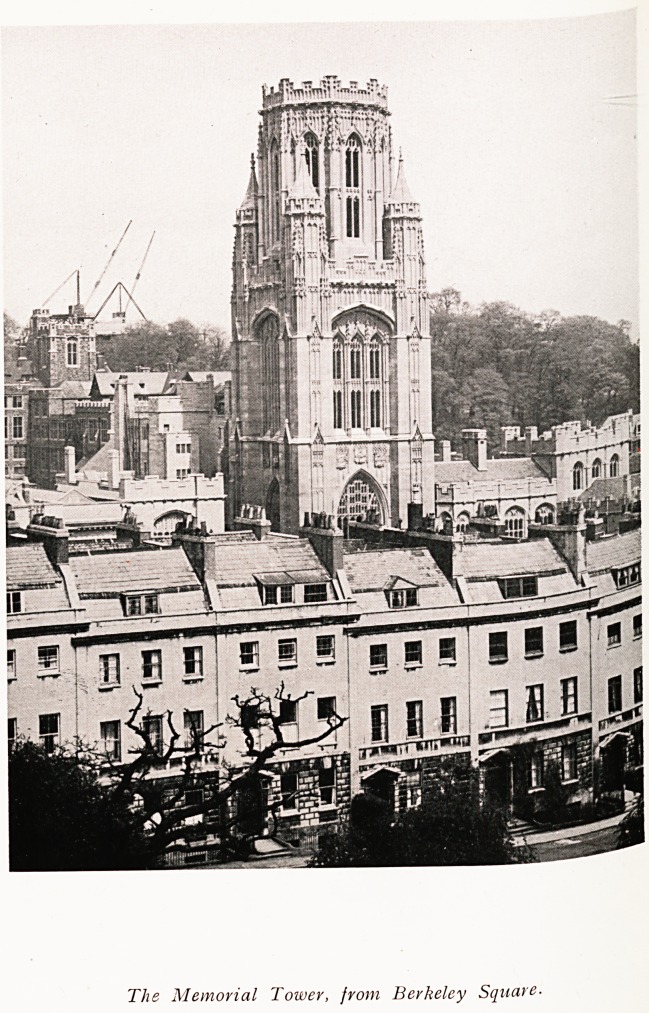# University of Bristol: Royal Opening of the New Buildings

**Published:** 1925

**Authors:** J. A. Nixon


UNIVERSITY OF BRISTOL.

THE MEMORIAL AND ARROWSMITH TOWERS WITH THE GROUNDS OF THE UNIVERSITY.

*Seen from the Physics Laboratory.*

### The Bristol Medico-Chirurgical Journal

*" Scire est nescire, nisi id me

Scire alius scierit."*

### UNIVERSITY OF BRISTOL.

### Royal Opening of the New Buildings.

On June 9th their Majesties the King and Queen will visit Bristol to open the new buildings of the University. The occasion will be a most notable one in the annals of the City and the University.

It is nearly three centuries since Dr. Dell, Cromwell's Chaplain, made the first suggestion that there should be a University in Bristol. His plan for provincial universities outside of Oxford and Cambridge met with no favour and has long been forgotten.

Higher learning met with no encouragement in Bristol until the decay of the Barber Surgeons Company in the early part of the eighteenth century stimulated some of the medical men on the Infirmary staff to give anatomical and other lectures for the benefit of their surgical pupils. In 1816 Dr. James Cowles Prichard conceived the project of founding a Medical School in connection with the Infirmary, " an institution which furnishes so many valuable opportunities for professional improvement." By 1830

various independent courses of lectures had been combined to form two recognised medical schools, viz. "The School of Anatomy and Medicine" in Limekiln Lane (recognised by the Apothecaries' Hall), and The Bristol Medical and Surgical School (recognised by the Royal College of Surgeons). A letter of Dr. Henry Clark's shows that in 1833 these two schools (or Anatomical Classes) were amalgamated to form "The Bristol Medical School."

Dr. Carrick delivered the inaugural address at the opening of this School on October 14th, 1833, and remarked at his conclusion that it appeared most desirable that a School of Medicine should form an integral part of the Bristol College. This idea seems never entirely to have been lost sight of by the teachers in the Bristol Medical School. When at last the University College was founded in 1877 some of the lecturers in the Medical School advocated complete incorporation of the School with the new College; but the teaching in the Medical School had been for some years previously of a low standard, and there was opposition from teachers who were reluctant to be brought under any critical supervision, so that the vote in favour of amalgamation was defeated. The Medical School remained autonomous with a mere "affiliation" to University College until 1892, when it was formerly "incorporated" in the College. Even after this "incorporation" the clinical part of the curriculum was managed independently of University College, and the students' fees for clinical instruction were paid directly to the staffs of the Royal Infirmary and General Hospital.

In 1909 King Edward VII. granted a charter to found the University of Bristol, whereby the University College and the Merchant Venturers' Technical College were merged into a new University. The incorporation of the Medical School was not even then made complete; the clinical part of the curriculum was still in fact a proprietary school in the hands of the staffs of the two medical institutions. This partial autonomy of the Medical School was, however, unsatisfactory and was unfavourably commented on by the Universities Grants Committee of the Treasury, so that in 1922 the teachers of clinical subjects in the Medical Faculty surrendered to the University their proprietary rights and the whole medical curriculum came under University management. Thus the Bristol Medical School with its long and honourable tradition continues as one of the most vigorous Faculties in the University to whose foundation it had so actively contributed, even if at times it has dissembled its filial affection and pride by a certain degree of independence.

Now that incorporation has been effectually carried out, and the seal of Royal approval is about to be set on the University and the new buildings which the munificence of the Wills family has called into being, it will not be inappropriate to review the opportunities and advantages which the medical student can enjoy to-day in Bristol.

The preliminary studies of Chemistry, Biology and Physics are carried on in some of the newest and most up-to-date laboratories in the kingdom. The Chemical wing of the University was built in 1911, and leaves little to be desired in the way of housing and equipment. The new Physics wing, built and endowed by the late Mr. H. H. Wills, is nearing completion close by the Royal Fort in Tyndall's Park. The intermediate subjects of Anatomy and Physiology have found quarters that would astonish the students of the old University College days. The dissecting room occupies the former Great Hall of the College facing the Grammar School field, and annexed to it are the Anatomy Theatre and Museum. The department of Physiology is situated in part of the wing by the Arrowsmith Tower ; the lecture theatre is large and admirably planned, but the laboratories are already too small to accommodate the large number of medical students. Above the Physiological theatre and laboratories is the Department of Pathology with its laboratories and museum. Pathology is a large and ever-growing department which provides not only for the instruction of students but also for an immense amount of pathological investigation for private doctors, public institutions, and municipal authorities in the surrounding counties as well as for the Ministry of Health (particularly as regards food poisoning outbreaks). The pathological museum is arranged on a new and most efficient plan. The departments of Medicine, Surgery, Obstetrics, Ophthalmology, Otology and Laryngology have a mere nominal habitat in a

*Pathological Museum.*

*Medical Library.*

small lecture room, and two professors' rooms adjoining the pathological museum. Although these departments find such scanty accommodation in the University buildings, they can spread their wings wider and more freely in the great hospitals of the City.

In the new buildings which the King will open in June there is, however, a fine Medical Library containing several collections of books which originally formed the separate libraries of the Royal Infirmary, the General Hospital, the Bristol Medico-Chirurgical Society and University College. This year the Medico-Chirurgical Society gave their whole library unreservedly to the University. Thus the Medical Library in our sixteen-year-old University can boast the possession of books which began to be collected into libraries nearly two hundred years ago, and contains many valuable old volumes and complete sets of periodicals which may well be envied by far older institutions.

The hospitals of the city furnish great wealth of clinical material which the management committees place gladly at the service of the University. The Bristol Medical School has always prided itself on the practical experience which its students can gain in clinical medicine. The number of cases available for each individual student is unsurpassed anywhere in the country. The Royal Infirmary and the General Hospital between them contain 630 beds, which are allotted as follows :—

| | B.R.I. | B.G.H. |
|---|---|---|
| Medical .. .. .. | 72 | 70 |
| Surgical .. .. .. | 126 | 92 |
| Gynæcological .. .. .. | 29 | 28 |
| Obstetric .. .. .. | 24 | 20 |
| Maternity cots.. .. .. | 24 | 12 |
| Ophthalmic .. .. .. | 9 | 4 |
| Ear, Nose and Throat .. | 16 | 14 |
| Children .. .. .. | 36 | 13 |
| Skin .. .. .. .. | 4 | 2 |
| Isolation, etc. .. .. .. | 22 | 13 |

For obstetric work each of these institutions has a large "district" where students attend midwifery cases in their own homes.

There are also Out-patient Clinics in every department, and in addition Venereal, Ante-natal and Infant Clinics. Two special hospitals (the Eye Hospital, 40 beds, and the Children's Hospital, 109 beds) have made their wards and out-patient departments available for University students.

The large modern Poor Law Hospital at Southmead (566 beds) has been opened to students by the City Guardians.

Instruction in infectious diseases is given at the City Fever Hospital, Ham Green (Fever Section 135 beds,

*Bristol Royal Infirmary.*

Sanatorium Section 136 beds), by arrangement with the City Health Committee and the Medical Officer of Health, and the same authorities admit students to study tuberculosis at the Municipal Dispensaries and Frenchay Park Colony for children (35 beds).

There is a small well-equipped Orthopædic Hospital where students are welcomed (36 beds).

*Bristol General Hospital.*

Special wards exist at Southmead Hospital for mentally deficient children (100 beds).

University courses in Mental Diseases and Insanity are given at the City Mental Hospital, Fishponds, by the Medical Superintendent.

Thus it will be seen that all the various authorities in Bristol responsible for hospital management have taken up the most friendly and helpful attitude towards the University and its medical students, both undergraduate and post-graduate.

An important feature in the curriculum is the amount of resident work permitted to unqualified students. Whilst acting as surgical ward dressers each student resides for one week in rotation as "resident dresser for the week." In this capacity the dresser sees all interesting emergencies, both in-patient and casualty, as well as assisting at immediate operations.

The midwifery course is exceptionally well-arranged. The student resides in hospital for six weeks; during the first two weeks he attends in the lying-in wards, and during the last month he visits the patients on the district, confining them in their own homes.

Postgraduate studies have been for some years well organised in the series of lectures by those of our clinicians who are most fitted to assist medical practitioners who feel the need for being made more efficient in the newer developments of medicine, surgery and pathology. The President of the Bristol Medico-Chirurgical Society, Dr. J. O. Symes, in his Presidential address, has contributed an authoritative article on the subject to this Journal.

The Colston Research Society has already done much to foster and by financial assistance to promote research in

*The Victoria Rooms, Clifton, now the Club Rooms of the University of Bristol Union.*

*The Memorial Tower, from Berkeley Square.*

connection with the University, and the scientific and clinical investigations in the realms of medicine and surgery are worthy to rank with the achievements attained in the other branches of science. The Society has earned the gratitude of the Medical Faculty, and needs only more generous support of the public benefactor to give further benefit in the evolution of medicine.

Apart from the instructional side of the curriculum Bristol is fortunate in being able to offer to undergraduates special residential advantages. Students who do not live at home are not now compelled to find isolated and ill-furnished lodgings.

For men there are two Halls of Residence: Mortimer House, Clifton, with accommodation for 35 students, and Canynge Hall, with accommodation for 78 students. A third hall is about to be built at Downside, Durdham Down, which will be designed on college lines in its own spacious grounds. For women two adjoining houses, Clifton Hill House and Callander House, together with the neighbouring Clifton Manor House, provide excellent accommodation in some of the finest mansions of Clifton. The University of Bristol has from the outset aimed at being a residential University, and in the short space of sixteen years has succeeded in laying the foundation of true collegiate life for both men and women students.

J. A. NIXON.

## Figures and Tables

**Figure f1:**
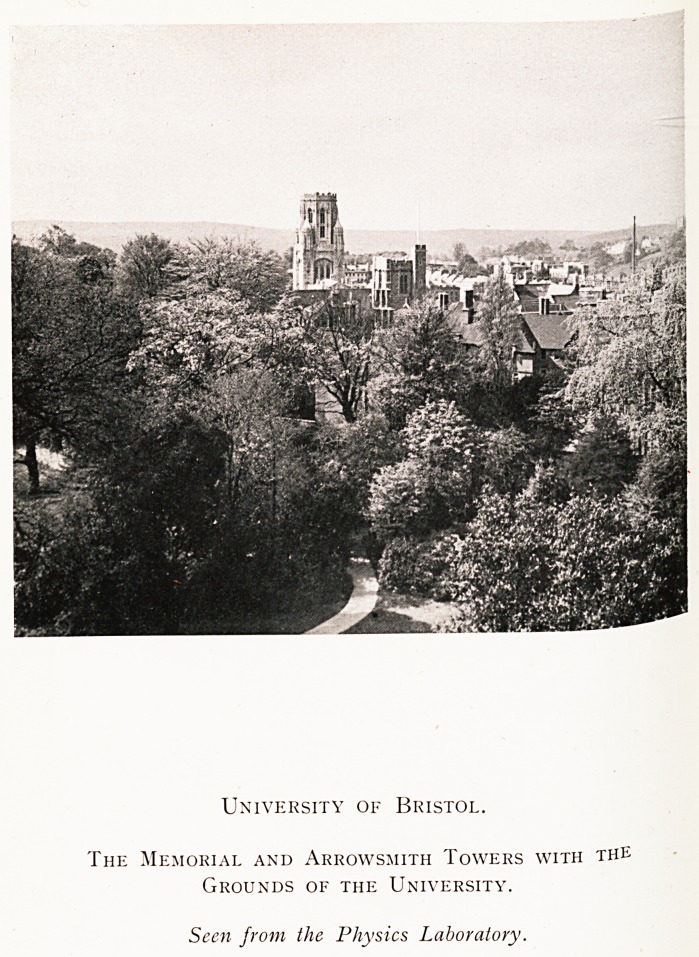


**Figure f2:**
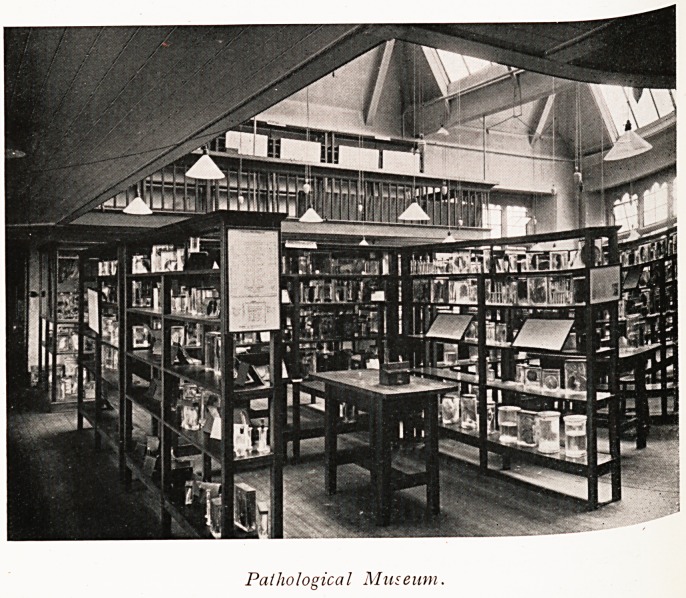


**Figure f3:**
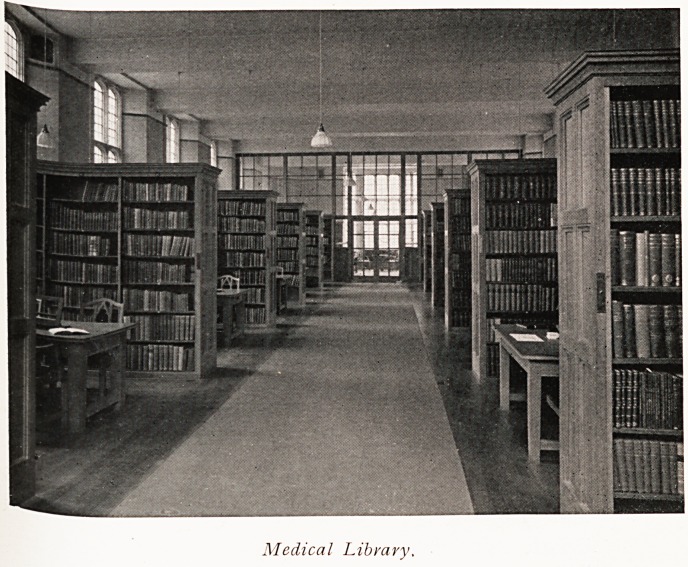


**Figure f4:**
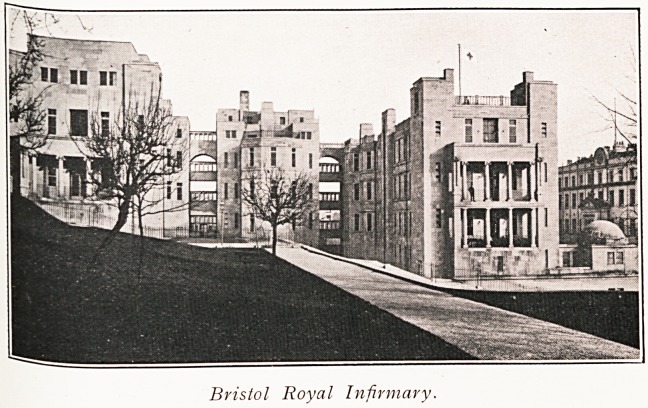


**Figure f5:**
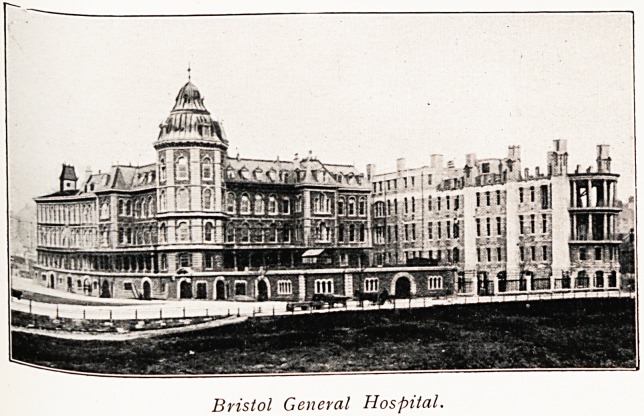


**Figure f6:**
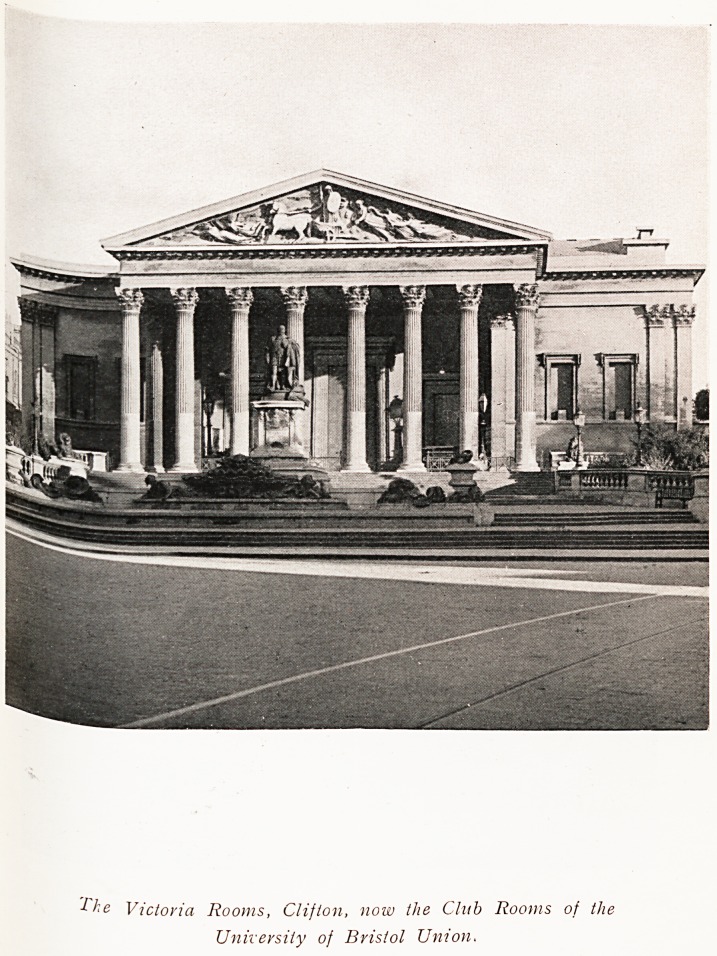


**Figure f7:**